# Reconocimiento y manejo endodóntico de primeros premolares maxilares con dos raíces y tres conductos. reporte de caso de bilateralidad

**DOI:** 10.21142/2523-2754-1102-2023-157

**Published:** 2023-06-29

**Authors:** Jairo Jhoel Molina Quispe

**Affiliations:** 1 División de endodoncia, Universidad Científica del Sur, Lima Perú. 100108745@cientifica.edu.pe Universidad Científica del Sur División de endodoncia Universidad Científica del Sur Lima Peru 100108745@cientifica.edu.pe

**Keywords:** primeras premolares maxilares, anatomía interna, bilateralidad, tratamiento endodóntico, maxillary first premolars, internal anatomy, bilaterality, endodontic treatment

## Abstract

La capacidad de reconocer las variaciones anatómicas del sistema de conductos radiculares es crucial antes de iniciar el tratamiento endodóntico. No menos importante es llevar a cabo estos tratamientos con base en la evidencia científica actual. Los premolares son las piezas dentales con mayor complejidad en su anatomía interna y las que se presentan en mayor porcentaje en comparación con otras piezas dentarias; si una pieza presenta anatomía accesoria, es altamente probable que la pieza contralateral presente la misma anatomía interna, a esto se le conoce como bilateralidad simétrica. Por lo tanto, las tomas radiográficas deberán ser ejecutadas con proyecciones proximales y ortorradiales para aproximarnos a la forma 3D que presentan estas anatomías y minimizar errores procedimentales. El uso de la magnificación es imprescindible para abordar todo el sistema de conductos y realizar un adecuado tratamiento endodóntico. El presente reporte de caso muestra el abordaje de los dos primeros premolares superiores maxilares de un mismo paciente con anatomía de tres conductos y simetría bilateral.

## Abstract (en)

The ability to recognize the anatomical variations of the root canal system is crucial before starting endodontic treatment, no less important is being able to carry out these treatments based on current scientific evidence. The premolars are the dental pieces with the greatest complexity in their internal anatomy and the ones that appear in the highest percentage compared to other teeth, if a piece presents accessory anatomy, it is highly probable that the contralateral piece presents the same internal anatomy, to this it is known as symmetrical bilaterality, therefore the radiographic images must be executed with proximal and orthoradial projections in order to approximate the 3D shape that these anatomies present and minimize procedural errors. The use of magnification is essential to be able to address the entire root canal system and perform an adequate endodontic treatment. This case report presents the approach to 2 maxillary first premolars from the same patient with 3-canal anatomy, presenting bilateral symmetry.

## INTRODUCCIÓN

El objetivo principal de la endodoncia es lograr el sellado hermético de los conductos radiculares a través de su limpieza y conformación. La presencia de un conducto no tratado puede llevar al fracaso ^1^.

La comprensión a profundidad de la anatomía interna del conducto radicular y sus variaciones en todos los grupos de dientes es sumamente necesaria para alcanzar el éxito en los procedimientos endodónticos, tanto quirúrgicos como no quirúrgicos. El concepto de una pieza con una raíz y un solo conducto radicular se debe considerar un error, al tener en cuenta que tenemos istmos, anastomosis, conductos accesorios y deltas apicales, razón por la cual en la actualidad se habla de sistema de conductos radiculares ^2^.

Los sistemas RVG son de ayuda para el diagnóstico; sin embargo, se ha demostrado que la tomografía computarizada *cone-beam* es más confiable para detectar algún canal extra ^3^, así como la delimitación de tejidos y lesiones periapicales ^4^.

Vertucci y Gegauff ^5^ reportaron la presencia de tres conductos con forámenes independientes en solo el 5% en un estudio de 400 primeros premolares maxilares descalcificados e inyectados con tinta. 

Las piezas maxilares tienden a presentar mayor simetría bilateral (número de raíces y configuración anatómica) que las piezas mandibulares. Específicamente, los primeros premolares superiores pueden presentar anatomía bilateral simétrica desde el 80% al 83,5% de casos ^6^^,^^7^. La Tabla 1 presenta la comparación de número de raíces y configuración anatómica interna de diferentes estudios con tomografía computarizada *cone-beam* en diferentes poblaciones, se puede apreciar que la prevalencia de tres raíces (0% al 2,6%) y la configuración tipo VIII de Vertucci (0% al 2,9%) es muy baja. 

**Tabla 1:** Prevalencia de número de raíces y configuración de conductos radiculares en primeras premolares según la clasificación de Vertucci

	Configuración del conducto según Vertucci (%)										
Estudio	Número de raíces (%)			I	II	III	IV	V	VI	VII	VIII
	1	2	3								
Bürklein *et al*. (^8^)	36,4	62,4	1,2	3,9	6,5	0,0	68,5	7,9	12,3	0,2	2,0
Tian *et al*. (^9^)	66	33,3	0,7	14	23	4	51	3	2	1	1
De Lima *et al*. (^10^)	18,2	80,2	1,6	6,5	7,7	0,6	82,2	0,8	0,6	0,0	1,6
Sabeer *et al*. (^11^)	45,8	53,1	1,1	1,1	15,6	1,4	73,2	1,4	2,9	1,7	1,7
Olczak *et al*. (^12^)	28,3	69,1	2,6	1,7	8,6	2,6	78,5	5,1	0,0	0,6	2,9
Al-Zubaidi *et al*. (^13^)	39,8	58,6	1,6	5,2	32,8	0,6	57,8	2,0	0,0	0,0	1,6
Haider *et al*. (^14^)	40	60	0,0	72	4	3	5	13	2	1	0,0
Aguilera *et al*. (^15^)	71,1	28,9	0,0	31,4	16,5	10	41,3	0,8	0,0	0,0	0,0
Abella *et al*. (^16^)	46	51,4	2,6	25,1	10,2	4,4	52,8	1,9	1,6	1,4	2,6
Cobos *et al*. (^17^)	74,5	25,5	0,0	30,2	17,9	7,5	33,1	2,8	6,6	1,9	0,0

El presente reporte de caso presenta el abordaje de los dos primeros premolares superiores maxilares de un mismo paciente con anatomía de tres conductos y simetría bilateral.

## REPORTE DE CASO

**Figura 1:**
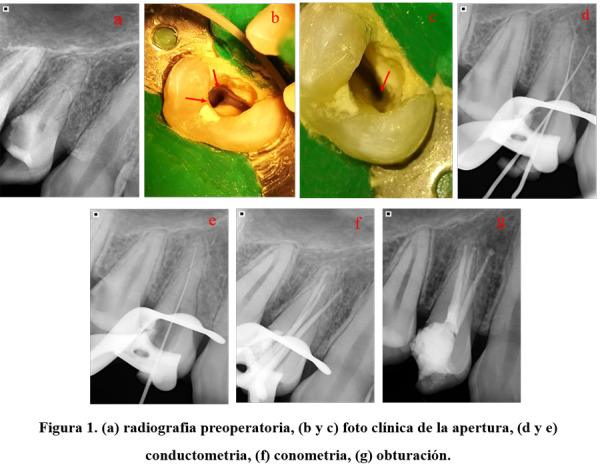
(a) radiografía preoperatoria, (b y c) foto clínica de la apertura, (d y e) conductimetría, (f) conometría, (g) obturación.

Paciente masculino de 21 años sin antecedentes médicos de importancia ASA 1, acude a la consulta por sensibilidad marcada al frío, con episodios de dolor espontáneo de duración aproximada de 30 segundos, localizado en la zona superior derecha e izquierda correspondiente al área premolar. Se realizó un test de sensibilidad pulpar en las piezas 14, 15, 24 y 25, y se tuvo respuesta positiva en todas las piezas, con la diferencia de que la sensibilidad fue persistente en las piezas 14 y 24. No se hallaron bolsas periodontales al sondaje. La radiografía de diagnóstico (Figs. 1a y 2a) reveló lesiones cariosas en caras distales, las cuales se encontraban en contacto con la pulpa dental. La zona periapical se apreciaba indemne con lámina dura sin alteraciones. El diagnóstico pulpar fue de pulpitis irreversible y diagnóstico periapical de periodonto sano en piezas 14 y 24. El examen radiográfico reveló un ancho excesivo en la raíz vestibular de las piezas 14 y 24; adicionalmente, la pieza 24 mostraba borramiento en la trayectoria de su conducto radicular a nivel del tercio medio-apical, lo cual despertó la sospecha de conductos extras. 

## DESCRIPCIÓN DEL CASO 1

Primera cita. Se procedió a retirar el tejido carioso en la cara distal de la pieza 14, se reconstruyó la pared con cemento a base de policarboxilato, se realizó aislamiento absoluto y se procedió con la apertura cameral con piedras diamantadas redondas, bajo magnificación con lupas de aumento 6x. Se localizó el conducto palatino y, posteriormente, el orificio del conducto vestibular, el cual se apreció bastante amplio. Con la ayuda de insertos ultrasónicos troncocónicos diamantados D4 (WoodPecker), se realizó la extensión hacia distal, donde nos percatamos de la entrada de un conducto, y se irrigó con hipoclorito de sodio al 2,5% (Figs. 1b y 1c) para observar el “efecto champagne”. Inmediatamente, se procedió a permeabilizar los conductos con limas k#10, se tomó la conductometría digital con la ayuda de localizador foraminal (ApexID, SybronEndo), y se corroboró con una radiografía distalizada para evidenciar la presencia del conducto vestibulodistal y vestibulomesial (Fig. 1d). Se aplicó lo mismo para el conducto palatino (Fig. 1e). Por motivos de tiempo, se realizó el sellado cameral con policarboxilato y se postergó el tratamiento para una segunda cita.

Segunda cita. Pasados cuatro días, se procedió a retirar el policarboxilato. La preparación biomecánica rotatoria continua de los conductos vestibulares, se realizó con ProTaper Gold siguiendo la secuencia del fabricante hasta el instrumento F3 (Dentsply Maillefer), el conducto palatino se instrumentó con R#40 Reciproc Blue (VDW), con la ayuda del motor VDW Silver Reciproc. El protocolo de irrigación constó de 20 ml de hipoclorito de sodio al 2,5% y 5 ml de EDTA al 17% (Maquira), como quelante. Se activó el irrigante con puntas de ultrasonido (WoodPecker) en 3 ciclos de 20 segundos por cada conducto. Seguidamente, se procedió a realizar la conometría con cono R#40 blue para el conducto palatino y conos F3 ProTaper para los conductos vestibulares (Fig. 1f). Se procedió a secar los conductos con conos de papel 30.04 y 40.04 (Spydent). Para la obturación, se usó cemento sellador hidráulico bioactivo NeoSealer Flo (Avalon), en los conductos vestibulares se empleó la técnica de cono único (F3) y para el conducto palatino se empleó la técnica de compactación lateral en frío (R#40 y conos accesorios 20.02). Se limpió la cámara pulpar con alcohol al 95% y se realizó el sellado cameral con policarboxilato. Finalmente, se tomó la radiografía de obturación (Fig. 1g) y se derivó para su rehabilitación. 

## DESCRIPCIÓN DEL CASO 2

**Figura 2:**
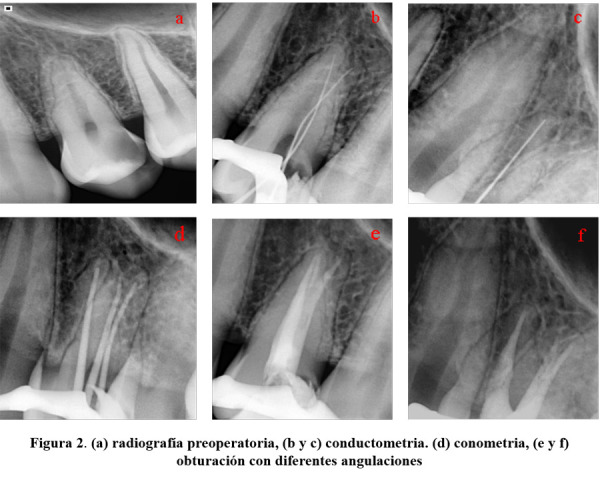
(a) radiografía preoperatoria, (b y c) conductimetría, (d) conometría, (e y f) obturación con diferentes angulaciones

Pasada la semana, se procedió a tratar la pieza 24. Se removió el tejido carioso con reconstrucción de la pared distal y se aisló el campo operatorio. La apertura cameral se realizó con piedras diamantadas redondas bajo magnificación 6x, y se apreció la configuración de la pieza 14. Se procedió a realizar la permeabilización de los conductos vestibulomesial, vestibulodistal y palatino; se tomó la conductometría digital con la ayuda de localizador foraminal (ApexID, SybronEndo) y la radiografía distalizada (Fig. 2b). Se aplicó el mismo procedimiento para el conducto palatino (Fig. 2c). A continuación, se realizó la instrumentación biomecánica rotatoria continua de todos los conductos hasta Mtwo 35.04 (VDW) accionado por el motor VDW Silver Reciproc. El protocolo de irrigación constó de 20 ml de hipoclorito de sodio al 2,5% y 5 ml de EDTA al 17% (Maquira). Se activaron los irrigantes con insertos ultrasónicos (WoodPecker) 3 ciclos de 20 segundos por cada conducto. Se secaron los conductos con conos de papel 35.04 (Spydent) y se realizó la conometría (Mtwo #35.04) (Fig. 2d). Posteriormente, se obturó con la técnica de compactación lateral con cemento sellador hidráulico NeoSealer Flo (Avalon). Se tomó la radiografía con angulación mesial y distal (Figs. 2e y 2f). Finalmente, se limpió la cámara pulpar con alcohol al 95% y se realizó el sellado cameral con policarboxilato.

## DISCUSIÓN

Como se puede apreciar en el reporte de caso, la disposición de las primeras premolares es parecida a la de una molar, por lo cual también ha recibido la denominación de “minimolar” ^18^. La presencia de una raíz se considera una variante anatómica común en el primer premolar maxilar ^19^ y se puede verificar desde la radiografía de diagnóstico en proyección ortoradial. Tal como lo indica Sieraski ^20^, si el ancho mesiodistal de la mitad de la raíz es igual o mayor que el ancho mesiodistal de la corona, es muy probable que se trate de premolares con tres raíces, aunque en algunos casos esta regla no se aplique a premolares con dos raíces y tres conductos, como se muestran las dos premolares presentadas en este reporte de caso, que se considerarían dentro de la configuración tipo VIII de Vertucci, la cual incluye las piezas con tres raíces separadas y tres conductos, dos raíces en la cual los conductos vestibulares se encuentra en la raíz vestibular fusionada y la raíz palatina solo con un conducto o tres raíces fusionadas con tres conductos ^21^.

Dentro del acto operatorio, si se observa que no existe una configuración clásica del piso de cámara, es decir, una relación lineal vestíbulo-palatina y en su lugar se aprecia un desplazamiento excéntrico en el plano mesiodistal de la raíz vestibular, es un indicativo de la presencia de tres conductos ^22^. El abordaje de esta anatomía es particular, debido a que se tendrá que modificar la apertura de la cámara pulpar en forma de una “T” para tratar todos los conductos ^20^. En algunos casos, la división del orificio de entrada de los conductos puede ubicarse un poco por debajo del piso de cámara, como en el caso 2 (1 o 2 mm en profundidad), lo cual podría dificultar el acceso de estos conductos; ante este caso se debería de instrumentar ultrasónicamente para extenderse de 1 a 2 mm apicalmente, lo que se podría considerar como un *preflaring* conservador. 

Como toda pieza con conductos adicionales, durante la preparación químico-mecánica se debe ser cuidadoso, ya que los conductos vestibulares podrían ser muy estrechos o curvos, lo cual podría llevar a errores procedimentales como perforaciones, escalones, fractura de instrumentos o bloqueo de algún conducto. Se aconseja el uso de instrumentos de níquel-titanio flexibles de baja conicidad, así como abundante irrigación con EDTA e hipoclorito de sodio ^23^. En la obturación, Almazrou ^24^ indica que podría bloquearse uno de los conductos vestibulares con un cono de papel y obturarlos individualmente, especialmente cuando se aplica la técnica de onda continua de calor y se realizará el *backfill*. También se podría emplear la técnica de compactación lateral o de cono único ^23^, esta última siempre con el uso de cementos selladores hidráulicos.

En la actualidad, el manejo de premolares con tres conductos se basa en el uso preoperatorio de tomografía computarizada *cone-beam* y el uso del microscopio operatorio o magnificación para el abordaje correcto de estos casos ^21^^,^^24^^,^^25^.

Es importante destacar que, según los estudios de prevalencia pertenecientes a Europa, Asia y una población africana ^8^^,^^9^^,^^11^^-^^14^^,^^16^ revelaron que la presentación de una configuración tipo VIII de Vertucci en primeros premolares es muy rara (0%-2,9%). En el caso de la población latinoamericana (Brasil, Chile, Perú, México y Colombia) es muy parecido ^10^^,^^15^^,^^17^^,^^26^^,^^27^, pues no superan el 2,6% de prevalencia; sin embargo, no debemos olvidar que existen y podrían presentarse en la clínica. La bilateralidad simétrica fue otra característica por resaltar, lo que confirma los hallazgos de Johnsen y Mashyakhy ^6^^,^^7^.

## CONCLUSIONES

Ante la sospecha de anatomía accesoria en alguna pieza candidata a un tratamiento de endodoncia, idealmente, debería tomarse una tomografía *cone-beam* y el manejo debería realizarse bajo magnificación, ya sea mediante lupas o microscopio; de esta forma se evita perder algún conducto extra y se puede abordar mejor el conducto. En el presente reporte de caso, se puede visualizar la presencia de las dos primeras premolares con dos raíces y tres conductos, algo poco habitual, por lo que la prevalencia de esta configuración se consideraría atípica. Además, se presentó con bilateralidad simétrica, es decir, si tratamos un diente con esta configuración, es altamente probable que la pieza contralateral presente la misma anatomía interna.

## Fuente de financiamiento:

Autofinanciado
